# Bridging the gap: A library‐based collaboration to enhance data skills for clinical researchers

**DOI:** 10.1002/lrh2.10339

**Published:** 2022-09-06

**Authors:** Matthew B. Carson, Sara Gonzales, Pamela Shaw, Daniel Schneider, Kristi Holmes

**Affiliations:** ^1^ Galter Health Sciences Library & Learning Center Northwestern University Feinberg School of Medicine Chicago Illinois USA; ^2^ Northwestern Medicine Enterprise Data Warehouse Northwestern Medicine and Northwestern University Feinberg School of Medicine Chicago Illinois USA; ^3^ Department of Preventive Medicine (Health and Biomedical Informatics) and Galter Health Sciences Library & Learning Center Northwestern University Feinberg School of Medicine Chicago Illinois USA

**Keywords:** clinical data warehouse, data science, learning health system, medical informatics, research data management, workforce development

## Abstract

**Introduction:**

Enterprise data warehouses (EDWs) serve as foundational infrastructure in a modern learning health system, housing clinical and other system‐wide data and making it available for research, strategic, and quality improvement purposes. Building on a longstanding partnership between Northwestern University's Galter Health Sciences Library and the Northwestern Medicine Enterprise Data Warehouse (NMEDW), an end‐to‐end clinical research data management (cRDM) program was created to enhance clinical data workforce capacity and further expand related library‐based services for the campus.

**Methods:**

The training program covers topics such as clinical database architecture, clinical coding standards, and translation of research questions into queries for proper data extraction. Here we describe this program, including partners and motivations, technical and social components, integration of FAIR principles into clinical data research workflows, and the long‐term implications for this work to serve as a blueprint of best practice workflows for clinical research to support library and EDW partnerships at other institutions.

**Results:**

This training program has enhanced the partnership between our institution's health sciences library and clinical data warehouse to provide support services for researchers, resulting in more efficient training workflows. Through instruction on best practices for preserving and sharing outputs, researchers are given the tools to improve the reproducibility and reusability of their work, which has positive effects for the researchers as well as for the university. All training resources have been made publicly available so that those who support this critical need at other institutions can build on our efforts.

**Conclusions:**

Library‐based partnerships to support training and consultation offer an important vehicle for clinical data science capacity building in learning health systems. The cRDM program launched by Galter Library and the NMEDW is an example of this type of partnership and builds on a strong foundation of past collaboration, expanding the scope of clinical data support services and training on campus.

## 1 INTRODUCTION

The modern learning health system (LHS) integrates internal data and experience with external evidence to put knowledge into practice.^1^ As a result, patients get higher quality, safer, more efficient care, and healthcare delivery organizations become better places to work. A mature LHS relies on people, systems, and data to capture evidence and drive continuous quality improvement. Data and technical systems play an essential role in a successful LHS, requiring a trained workforce to drive research and strategic activities and realize continuous quality improvement.

## 2 QUESTION OF INTEREST

Several challenges complicate an LHS from fully realizing the potential of investments in digital health infrastructure, including workforce readiness, sharing and access of data and research outputs, social and domain silos, challenges with research reproducibility and reuse, and other interdisciplinary capacity building efforts.^2^ There is also a need to improve communication between clinical researchers and technical staff by introducing technical concepts and vocabulary to researchers in preparation for collaboration. Addressing both the social and technical aspects of the process further advances data reuse and discovery. Here, we describe key efforts to bridge these gaps through a clinical research data management (cRDM) program for researchers, developed in partnership by Galter Library and the Northwestern Medicine Enterprise Data Warehouse (NMEDW).^3^, ^4^

## 3 METHODS

### 3.1 The Northwestern Medicine Enterprise Data Warehouse

**FIGURE 1:**
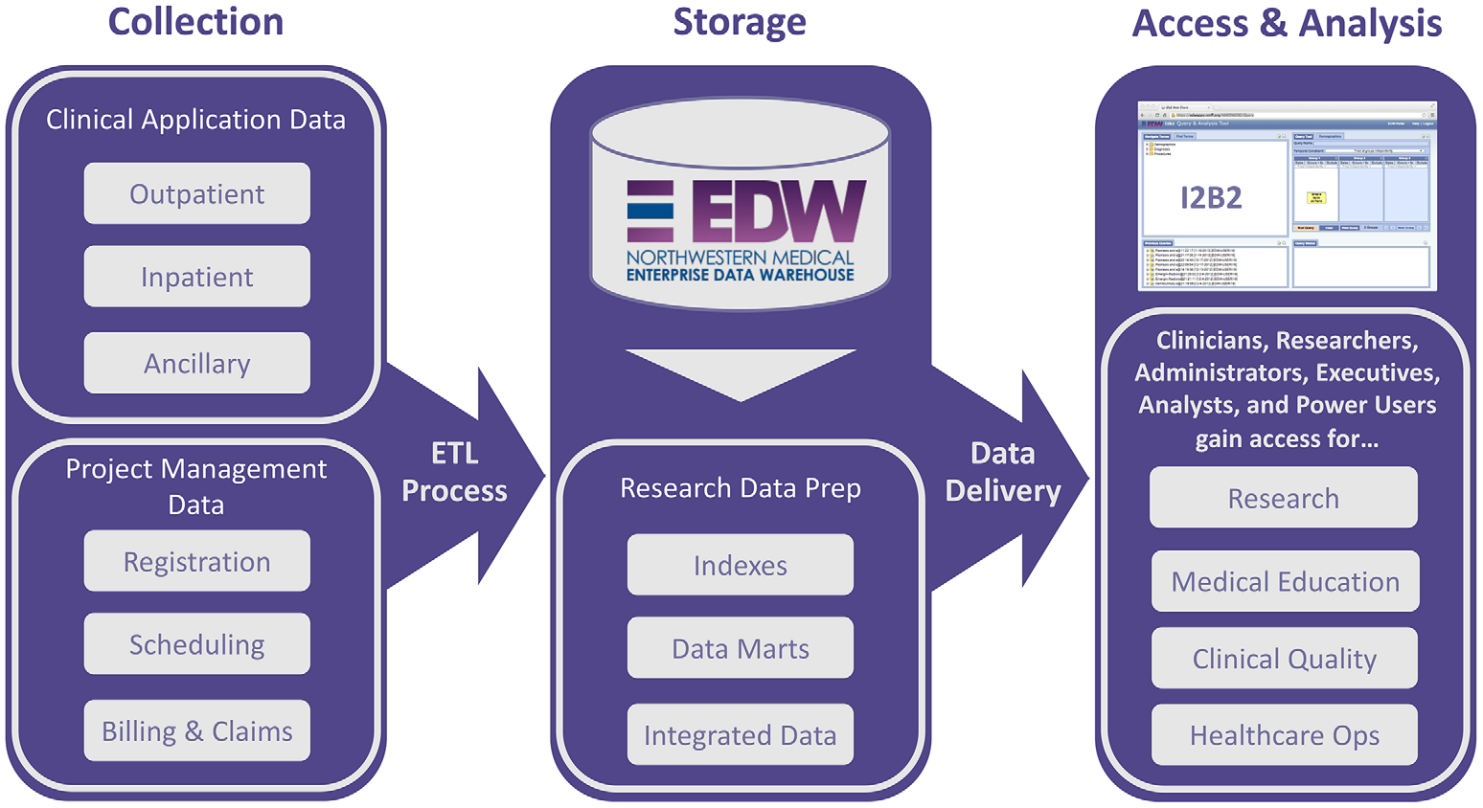
Data from Northwestern Medicine electronic health record systems are copied to the Northwestern Medicine Enterprise Data Warehouse via a nightly extraction, transformation, and loading (ETL) process. Data integration is performed where possible and indexes and data marts are created to support research requests. Feasibility analysis can be conducted using the Informatics for Integrating Biology and the Bedside (i2b2) tool, and a variety of clinical and translational personnel can request access for research, medical education, clinical quality studies, or healthcare operations analysis.

The NMEDW is a highly integrated, massively scaled, highly secure centralized database repository containing clinical, financial, operational, and regulatory data on patients who receive their care from the Northwestern family of healthcare providers. It is a comprehensive and integrated repository of all clinical and research data sources on the campus to facilitate research, clinical quality, healthcare operations, and medical education. The NMEDW currently stores observations on more than 9.2 million distinct patients. Each day, it loads more than 2.8 billion new data elements from 142 separate sources, including electronic health records, pathology data from the hospital and research laboratories, biomarker data from research databases, and research transactional data from our electronic institutional review board (eIRB) and other institutional systems. It has a mature data steward model, supported by an electronic workflow that enables rapid review of data requests (Figure 1).

The NMEDW can be leveraged for research purposes through a variety of resources and processes. Self‐service exploration of cohort availability is facilitated through the i2b2 Feasibility Query Tool. Likewise, individuals who have demonstrated sufficient skill with databases by passing an exam can apply to become a “Power User” and be granted direct access to the NMEDW for research or quality‐improvement purposes. Individuals who need more support can work with the data analyst team for a fee. However, capacity for analyst time is severely limited, due to increased enhancement and integration of this critical resource into campus and clinical workflows, as well as significantly expanded scope and overall demand for NMEDW data in research.

### 3.2 The Galter DataLab

When well integrated with Clinical and Translational Science Award institutes, health sciences libraries can provide an effective and efficient sociotechnical support structure for research. In Northwestern University's Feinberg School of Medicine, the Galter Health Sciences Library (Galter) provides a variety of support services for faculty, staff, and students, and partners on several research projects with the Northwestern University Clinical and Translational Sciences Institute (NUCATS) and across the university. Embedding the library in research activities promotes more efficient identification of needs and streamlines efforts to address these through resources, services, and training. The DataLab research support core develops and designs novel, sustainable data services throughout all stages of the research data life cycle for faculty, staff, and students in the Feinberg School of Medicine.^5^ The DataLab provides a complimentary consultation service, the DataClinic, which follows a primary care model for data‐related consultation. Through this service the DataLab team works to resolve issues that fall within their expertise and link researchers with experts on campus who can help to resolve other issues. Engagement with the research community occurs through teaching, hosting, and sponsoring training events and workshops that promote best practices related to data management, reproducibility, compliance with data sharing policies, and open science, as well as a range of associated technical topics. The program discussed in this paper was developed within this support core.

### 3.3 A research data management program for clinical researchers

**FIGURE 2:**
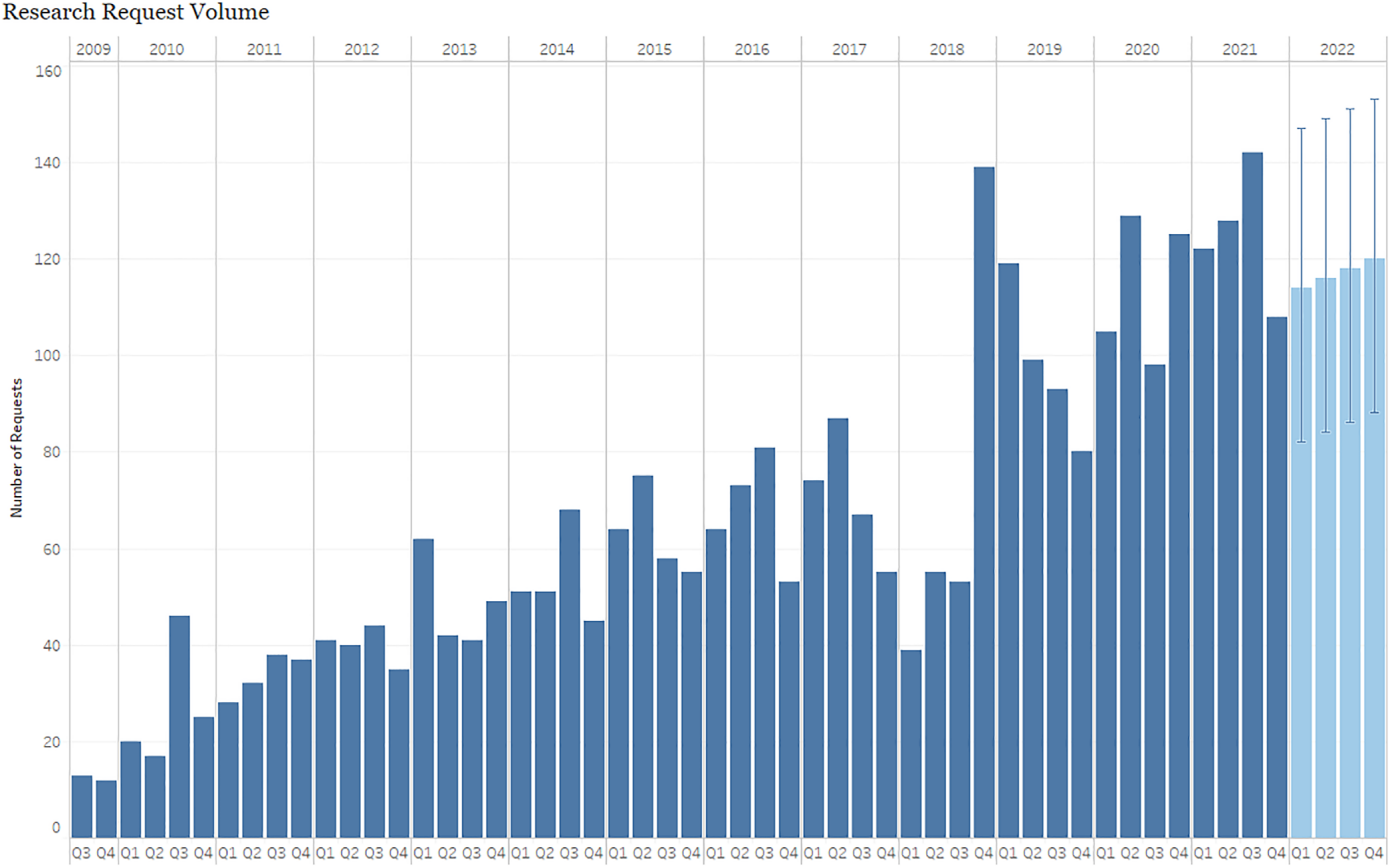
The number of research requests submitted to the Northwestern Medicine Enterprise Data Warehouse (NMEDW) over the last 12.5 years are shown, along with the projected number of requests over the next 12 months. Data provided by the NMEDW.

The NMEDW has seen an exponential increase in demand for new requests and this demand is projected to increase over the next 12 months (Figure 2). This increased demand for clinical research data highlights the need for increased local capacity though a comprehensive training program for system users. At the same time, resource constraints have made it difficult to provide training for clinician researchers that either helps to close the communication gap between these researchers and the data analysts or allows them to work directly with the data themselves. For early career researchers who are applying for funding and collecting and analyzing data, this gap in training can be a significant hurdle. Moreover, resulting research reports, which include important information such as project metadata, inclusion and exclusion criteria, structured query language (SQL) queries, and data plots, are often not stored in ways that support reuse and preservation, making it difficult for the larger research community to discover and explore previous projects that may help them realize efficiencies and advance their own work.

With proper training, researchers can translate their domain knowledge more effectively to their analyst partners, and vice versa. This improved communication pathway will not only increase efficiency but also help to avoid miscommunication that causes delays and can even result in incomplete or incorrect inclusion criteria for research cohorts. These improvements benefit the NMEDW with more efficient workflows and less one‐on‐one analyst time needed; the researchers, with an easier research startup process and a foundation of training and reference studies on which they can build their work; the university, which will save time and research funding that could be applied elsewhere; and the larger community, as project findings and resources are made openly available to others who seek to support this critical need for their LHS.

A targeted project was launched by Galter and NMEDW, building on a strong foundation of past discussion and collaboration, to create a cRDM training program (Table 1).

**TABLE 1:** Key goals of the clinical research data management program

Guide clinical researchers through the process of retrieving, collecting, and storing data from an EDW Promote improved communication and collaboration between data analysts and clinical researchers to make them better partners in research projects Enhance reusability of clinical reports and database queries by creating workflows and training for preservation of these research outputs and for discovery by the larger research community, both institution‐wide and beyond

## 4 RESULTS

### 4.1 Workshops and sandbox resources for cRDM training

**FIGURE 3:**
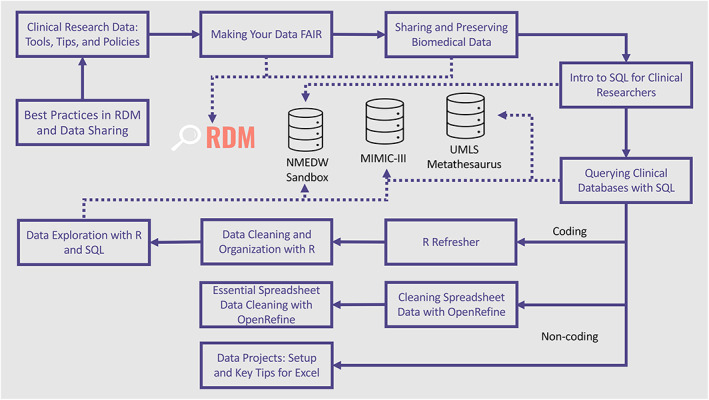
Workshops and supporting materials for the clinical research data management (cRDM) program cover a variety of topics including technical training, federal and local data policies, and best practices in data sharing and preservation. To support participants with a variety of skill levels and backgrounds, training is supplemented by university‐supported workshops, self‐paced online learning, and additional resources offered by Northwestern University IT Research Computing Services and others. cRDM content is updated based on evaluation and feedback from our training partners and participants. For links to current workshop content, see https://galterdatalab.github.io/crdm‐training/pages/training.html

A training curriculum designed to meet needs of clinical researchers is the foundation of the cRDM program (Figure 3). The curriculum features new workshops as well as existing training materials and guides that have been updated and improved to address key aspects of clinical research through an EDW, federal and local data policies, and best practices in data sharing and preservation. Figure 3 illustrates the cRDM training curriculum courses in a sequential manner for how they may be consumed, and how they relate to major EDW topics. Participants are also encouraged to use supplementary guides, summaries, and code developed for courses. Generally, participants engage in approximately 12‐15 hours of workshop instruction. Ultimately, self‐study and practice time varies depending on the experience level of the participant. As such, the program is designed to be taken over the course of a year and training is offered frequently to accommodate busy schedules. To promote improved communication and collaboration between data analysts and clinical researchers, NMEDW leadership and analysts serve as advisors for workshop development on both topics and content. This allows the team to request training program modifications that address any issues that arise during their collaboration with the researchers.

### 4.2 Enhancing preservation and discovery of research objects

To enhance reusability of NMEDW research reports, we are creating workflows and corresponding training for preservation and discoverability in our institutional repository, a next‐generation research data management system developed through an international collaboration with the European Organization for Nuclear Research (CERN) and other partners, called InvenioRDM.^6^, ^7^ InvenioRDM enables organizations to securely house research products and make them discoverable, shareable, reusable, and citable. It simplifies the process of collecting, preserving, and disseminating a wide range of research products that enhance individual and institutional visibility, promotes people and their expertise, enables discovery and accessibility by the international scientific community, and fosters open and FAIR science.^8^ It includes a wide range of features that can help power biomedical research through improved data sharing, knowledge dissemination, and interdisciplinary collaboration. Development on this platform is driven by user needs and informed by best practices and standards, including those that help define nedxt‐generation repositories as a foundation for a distributed, globally networked infrastructure for scholarly communication, discovery, and innovation.^9^ Since the system is designed to work with any research output type and can also be integrated with third‐party tools such as GitHub^10^ and Jupyter Notebooks, preservation of NMEDW research reports is an ideal use case for this system. This work is part of our orchestrated efforts to enrich the sociotechnical environment at Northwestern through projects to improve communication and training for the clinical and translational workforce,^11^, ^12^ properly attribute contributors of research outputs, enhance data practice and security workflows, and incorporate standards and persistent identifiers throughout. Some cRDM workshops (notably “Making Your Data FAIR” and “Sharing and Preserving Biomedical Data”) incorporate InvenioRDM to introduce best practices in data sharing.^9^ However, instruction could be generalized to other repository platforms, provided they include best‐practice features, such as those recently recommended by the White House Office of Science and Technology Policy.^13^, ^14^

### 4.3 Transparency and openness of training materials to promote sustainability

We make our work openly available to provide a template for clinical research data education and training for other institutions and enable feedback on the material for future improvements. All course materials, code, and documentation are freely available to the research community. Workshops are shared in online platforms such as GitHub so that our experience can provide a template for clinical research data training.^15^ To better support sustainability of this effort; ensure transparency, reproducibility, and security; and help advance program goals for this work to serve as a blueprint of best practice workflows for clinical research data training, we apply best practices in open science wherever possible (Table 2).

**TABLE 2:** Transparency and openness in training

Open access: All training and workshop materials are made openly available through open source repositories (GitHub) and as a collection in the institutional repository; published works will be submitted to open access journals.
Open platforms: Open architectures are used for all project activities, including GitHub, REDCap, and institutional repositories (here, InvenioRDM).
Open knowledge sharing: Train‐the‐trainer sessions will be held to train additional staff, with sessions recorded to support just‐in‐time needs, regardless of location.
Proper content and data stewardship by applying FAIR principles: By making our methods, training materials, and evaluation data findable, accessible, interoperable, and reusable, we encourage others to adopt our content and make customizations and improvements.
Promoting proper stewardship of knowledge: By making Northwestern Medicine Enterprise Data Warehouse research reports and SQL queries accessible and citable through InvenioRDM, clinical researchers will be able to benefit from past work for years to come.
Transparency: By making our processes, workflows, and results transparent through open access tools, we encourage others to build on our successes and find ways to improve on our less successful outcomes.

### 4.4 Staffing, engagement, and evaluation

The program is staffed and supported by the Galter Library DataLab, a team which consists of one data librarian, one bioinformatics and biosciences librarian, and one senior data scientist. DataLab programs, services, and resources are incorporated into their ongoing positions and scope of responsibilities. As such, there is no additional financial compensation for the consultation, although the cRDM program and other activities create opportunities for collaboration on research projects, many of which result in publications and grant support. Consultations may result in referrals to core facilities on campus with chargeback models that require compensation for more in‐depth services.

The participant population ranges from early‐career trainees to more established investigators and is predefined through ongoing work with NUCATS and the NMEDW, as well as through the education and training activities at Galter. This program is promoted through recurring on‐campus events and communication channels, and by Galter liaison librarians. While not compulsory at this point, the training suite offers a strong foundation of training for clinical researchers, critical to developing the cadre of investigators needed to support translational research. Toward this capacity goal, the cRDM program is made available to NUCATS trainees and trainees in other programs. This program and all other resources and services in the DataLab are supported by Feinberg School of Medicine through the library. Additional growth and program innovation will be supported internally or with grant support. The partnered approach by the DataLab and NMEDW teams has bolstered communication and collaboration directly between the two groups, leading to new and expanded partnerships on other projects.

Evaluation for the program is carried out in partnership between Galter and NUCATS to assess knowledge transfer and satisfaction, collaboration, communication, and outputs. For example, to assess the training, we use quantitative measures (eg, number of training sessions, volume of trainees, and career stage and specialty of trainees) and qualitative assessment (eg, pre‐/post‐event surveys to assess knowledge transfer and satisfaction and gather user success stories and feedback), all of which help inform subsequent strategies and directions. Since May 2020, 435 participants have completed a cRDM class, nearly all of whom participated during the development of the training series components. Upon completion of the training activity, participants completed a survey to assess satisfaction with training. Among survey respondents, 94% somewhat or strongly agreed that they learned a new skill in their training, and 92% planned to start using at least one resource or tool they learned about during the training and planned to share that tool with others. In addition, 89% said that the training session introduced them to at least one health information resource or tool that they had never used before. Assessment of the full impact of this program is a challenge, given the short duration to this point. With completion of program components, a more formal evaluation can now be undertaken. In their LHS logic model, Allen and colleagues highlight key outcomes to explore such as time and scale of adoption of evidence‐based practices, reductions of inefficiencies, improved health of the population, experiences of the patients and care teams, return on investment, and impacts on equity.^16^ These areas provide additional direction to understand true impacts of collaborations like the cRDM Program.

## 5 DISCUSSION

Library‐based partnerships to support training and consultation offer an important vehicle for clinical data science capacity building on campus. The cRDM program launched by Galter Library and the NMEDW is an example of this type of partnership and builds on a strong foundation of past collaboration, expanding the scope of clinical data support services and training on campus. Through training, clinical researchers gain essential clinical data skills for collecting, retrieving, and storing data from an EDW and incorporation of FAIR practices into research workflows to improve the reusability of research reports. Improved collaboration between clinical researchers and data analysts supports more effective partnerships. The program also focuses on sustainability, making materials and code discoverable and openly available for reuse and clinical research data education by the larger research community. Future work includes further enhancement of materials and delivery, packaging of the cRDM materials and a best‐practices guide for reuse by other sites, evaluation efforts to better understand downstream impacts of this program in the context of the LHS, and dissemination of the project to library and informatics communities through conference presentations and workshops.

It is important to note that there have been previous efforts in this space that have raised several important questions regarding incentives, sustainability, flexibility, and scalability of a program such as ours.^17^ We designed the cRDM program to respond to the evolving needs of our research environment by maintaining close communication with the campus community through NUCATS, the NMEDW, and liaison librarians. Even so, there remain opportunities for improvement across the program. We will continue to seek out prospects for continuous improvement and further alignment of DataLab and Galter Library resources, programs, and services with the needs and priorities of our campus.

## AUTHOR CONTRIBUTIONS

Matthew B. Carson and Kristi Holmes prepared the original draft of this paper. Matthew B. Carson, Sara Gonzales, Pamela Shaw, Daniel Schneider, and Kristi Holmes contributed to the methodology used. Matthew B. Carson, Sara Gonzales, and Kristi Holmes contributed to project administration. Matthew B. Carson, Sara Gonzales, and Daniel Schneider contributed to resource and code development. All authors were involved in reviewing and editing the manuscript.

## FUNDING INFORMATION

Developed resources reported in this publication are supported in part by the Northwestern Medicine Enterprise Data Warehouse, by the National Library of Medicine, National Institutes of Health (NIH) under cooperative agreement number 1UG4LM012346, and by the NIH's National Center for Advancing Translational Sciences grant number UL1TR001422. The content is solely the responsibility of the authors and does not necessarily represent the official views of the National Institutes of Health.

## CONFLICT OF INTEREST

The authors have no conflict of interests to report.

## Data Availability

The data underlying this article are available in the article.
